# Temperature-Sensitive Aerogel Using Bagasse Carboxylated Cellulose Nanocrystals/N-Isopropyl Acrylamide for Controlled Release of Pesticides

**DOI:** 10.3390/polym15224451

**Published:** 2023-11-17

**Authors:** Ni Dong, Zuzeng Qin, Wang Li, Nian Xiang, Xuan Luo, Hongbing Ji, Zhiwei Wang, Xinling Xie

**Affiliations:** 1 School of Chemistry and Chemical Engineering, Guangxi Key Laboratory of Petrochemical Resource Processing and Process Intensification Technology, Guangxi University, Nanning 530004, China; 15071850115@163.com (N.D.); qinzuzeng@gxu.edu.cn (Z.Q.); lw18372878434@126.com (W.L.); 13364048244@163.com (N.X.); luoxuan@gxu.edu.cn (X.L.); jihb@mail.sysu.edu.cn (H.J.); 2 Fine Chemical Industry Research Institute, School of Chemistry, Sun Yat-sen University, Guangzhou 510275, China; 3 Key Laboratory of Clean Pulp & Papermaking and Pollution Control of Guangxi, College of Light Industrial and Food Engineering, Guangxi University, Nanning 530004, China; wangzhiwei@gxu.edu.cn

**Keywords:** bagasse carboxylated cellulose nanocrystals, ammonium persulfate one-step oxidation method, temperature-sensitive aerogel, pesticide controlled release

## Abstract

Temperature-sensitive carboxylated cellulose nanocrystals/N-isopropyl acrylamide aerogels (CCNC-NIPAMs) were developed as novel pesticide-controlled release formulas. Ammonium persulfate (APS) one-step oxidation was used to prepare bagasse-based CCNCs, and then the monomer N-isopropyl acrylamide (NIPAM) was successfully introduced and constructed into the temperature-sensitive CCNC-NIPAMs through polymerization. The results of the zeta potential measurement and Fourier infrared transform spectrum (FTIR) show that the average particle size of the CCNCs was 120.9 nm, the average surface potential of the CCNCs was −34.8 mV, and the crystallinity was 62.8%. The primary hydroxyl group on the surface of the CCNCs was replaced by the carboxyl group during oxidation. The morphology and structure of CCNC-NIPAMs were characterized via electron microscopy, X-ray diffraction (XRD), X-ray photoelectron spectroscopy (XPS), compression performance, porosity analysis, and thermogravimetric (TG) analysis. The results demonstrate that CCNC-NIPAM has a high porosity and low density, as well as good thermal stability, which is conducive to loading and releasing pesticides. In the swelling, drug loading, and controlled release process, the CCNC-NIPAM exhibited significant temperature sensitivity. Under the same NIPAM reaction amount, the equilibrium swelling rate of the CCNC-NIPAM first increased and then decreased with increasing temperature, and the cumulative drug release ratio of the CCNC-NIPAM at 39 °C was significantly higher than that at 25 °C. The loading efficiency of the CCNC-NIPAM on the model drug thiamethoxam (TXM) was up to 23 wt%, and the first-order model and Korsmyer–Peppas model could be well-fitted in the drug release curves. The study provides a new method for the effective utilization of biomass and pesticides.

## 1. Introduction

As the most abundant natural material, cellulose has the characteristics of being renewable, biodegradable, non-toxic, abundant reserves, biocompatible, low cost, and so on. Bagasse is an important by-product of the sugar industry, and in recent years, the global annual production of bagasse has reached 260 million tons [1]. How to recycle the large amount of cellulose contained in bagasse has become a major research issue for many researchers [2]. Cellulose exists in bagasse in the form of nanoscale microfibers, arranged in larger fiber aggregates and fiber structures [3]. The surface hydroxyl groups and relatively large specific surface area provide abundant active sites for nanocellulose modification [4]. In addition to the advantages of cellulose, carboxylated cellulose nanocrystals (CCNCs) have a higher specific surface area, and their surface carries a negative charge to bind more types of drugs through electrostatic action [5], which provides more possibilities for its application in the field of drug controlled release preparations.

The preparation process of CCNCs focuses on reducing the size of cellulose, removing the amorphous region in cellulose as far as possible [6,7,8], and modifying the primary and secondary hydroxyl groups on the surface of cellulose to improve the miscibility and interfacial compatibility of the product CCNCs with solvents and give it new functions [9,10]. The common preparation principle is to directly oxidize the primary hydroxyl group on the surface of the cellulose. After modifying the primary hydroxyl group on the surface of the cellulose with a negative charge, –COOH, the surface of the product CCNCs forms a double-electric-layer structure with a certain thickness in water and achieves stable dispersion in the water phase by electrostatic force. According to the types of oxidants used, the preparation methods can be divided into TEMPO mediation [11,12], ammonium persulfate (APS) oxidation, and potassium permanganate oxidation. Among them, TEMPO oxidation and potassium permanganate oxidation require relatively high original cellulose purity; that is, the use of these two methods to prepare CCNC requires the prior removal of non-cellulose components of raw natural biomass, such as lignin, hemicellulose, and pectin. Complex processing steps and highly toxic chemical agents hinder the use of both methods. APS is a low-toxicity, high-water-solubility, and low-cost oxidizer. The APS oxidation degradation of cellulose is a simple and common one-step method. After persulfate dissolution, free radicals and hydrogen sulfate are generated, which can not only enter and degrade the amorphous region of cellulose but also degrade lignin, thus obtaining CCNCs with a high crystallinity and high oxidation degree. Some researchers have used a variety of biomass raw materials to prepare high-activity CCNCs through APS one-step oxidation and proved that this method is easy to produce on a large scale [13].

To deal with the threat posed by the overuse of pesticides to the environment and human beings, researchers have proposed pesticide controlled release preparations as a solution. Most pesticide active ingredients are hydrophobic organic compounds that require the addition of carriers, solvents, emulsifiers, dispersants, or other auxiliary ingredients to make them easy to use in the field. Thiamethoxam (TXM) belongs to the second generation of nicotinic insecticides, which includes a wide range of insecticidal species that have tactile and gastric toxic effects on pests, high stability, and are easily decomposed. It was used as a model drug in this study.

Aerogel is a kind of three-dimensional nanostructured porous material composed of nanoparticles or polymer molecular chains that has the structural characteristics of low density, high porosity, high pore volume, and high specific surface area. Environmentally responsive aerogels can stimulate and respond to changes in the environment such as light, pH, and temperature and are widely used as carriers for drug controlled release preparations [14]. Researchers have developed a variety of temperature-sensitive gels [15,16] whose components contain functional polymers such as N-isopropyl acrylamide (NIPAM) [17], chitosan, and hyaluronic acid [18]. Poly (N-isopropyl acrylamide) (PNIPAM) is a polymer composed of the hydrophilic amide group and a hydrophobic isopropyl side chain. It has the characteristics of temperature sensitivity, low toxicity, and easy chemical modification. When the temperature is lower than Lower Critical Solution Temperature (LCST), the amide group in PNIPAM interacts strongly with the water molecules to form a uniform polymer solution. When the temperature is higher than LCST, the interaction between isopropyl side chains in PNIPAM is enhanced, and the force between the amide groups and water molecules is weakened, thus forming a gel. The LCST of PNIPAM is generally 32 °C, which is close to the temperature of the actual use environment. Moreover, research on the composite gel based on PNIPAM can also improve its biocompatibility, mechanical strength, and drug loading.

To prepare pesticide controlled release preparations with ideal mechanical and drug release properties, the physicochemical properties of the complexes can be improved by adding nano-additives to the polymer skeleton [19]. Due to their good biocompatibility and high specific surface area, CCNCs have been used as a biological material for pesticide delivery [20], which can provide the possibility of high specific drug loading. The negative charge carried on the surface of CCNCs enables them to combine with various water-soluble drugs through electrostatic action. In addition, appropriate chemical or physical modifications to CCNCs can increase the variety of drugs that bind to CCNCs and promote the release of different drugs through different mechanisms.

In this study, bagasse was used as a raw material to prepare CCNCs through APS one-step oxidation, and then NIPAM was introduced. Under the action of a crosslinking agent and initiator, a temperature-sensitive nanocellulose aerogel (CCNC-NIPAM) was formed. The morphology and structure of the CCNC-NIPAM were analyzed with Fourier transform infrared (FTIR) spectroscopy, X-ray diffraction (XRD), thermogravimetric (TG) analyses, and electron microscopy. A single-factor experiment was designed to adjust the structure and properties of the gel. TXM was used as the model drug to study the performance of the aerogel in the subsequent drug loading and controlled release experiment. In addition, the existing zero-order model, first-order model, Higuchi model, and Peppas model were used to fit the release curves to explore the drug release mechanism.

## 2. Materials and Methods

### 2.1. Materials

Bagasse was obtained from Guangxi, China (main composition and content: lignin 18–25 wt%, cellulose 50–55 wt%, hemicellulose 15–30 wt%, industrial impurities about 7 wt%.). Ammonium persulfate (APS, Analytical Reagent) was purchased from Guangdong Guanghua Technology Co., Ltd. (Shantou, China). N-isopropyl acrylamide (NIPAM, ≥95 wt%, including stabilizer MEHQ) and thiamethoxam (TXM, ≥95 wt%) were purchased from Macklin Co., Ltd. (Shanghai, China). N, N′-methylene diacrylamide (Chemically Pure) and phosphate buffered saline (PBS) (analytical reagent) were purchased from the Sinopharm Group (Shanghai, China). All chemicals used in this study were used after receipt and were not further purified.

### 2.2. Preparation of CCNCs

Bagasse was put into a planetary ball mill tank for ball milling treatment for 20 min; 1.5 g of powder was weighed and added into 150 mL of APS solution, and then stirred at 500 r/min at 60 °C. After the reaction for several hours, the product was centrifuged several times at 10,000 r/min until the supernatant was a stable light blue CCNC suspension. The precipitation and supernatant after centrifugation were retained, and an ultrasound was performed after appropriate dilution. The bulk precipitates were dispersed and put into dialysis bags for 3 days. The supernatant was kept and stored in a refrigerator at 4 °C for later use. Some of the suspension was lyophilized to obtain CCNCs in powder form.

### 2.3. Preparation of CCNC-NIPAM

A certain amount of CCNCs and NIPAM was weighed and dissolved in deionized water. N, N′-methylene diacrylamide (MBA) was used as the crosslinking agent, and potassium persulfate was used as the initiator. The mixture was evenly mixed under the protection of N_2_. Three hours after the reaction, 200 mL of pre-cooled deionized water was added to the flask to terminate the reaction, and the solvent was changed periodically to remove the unreacted monomer. After the solution was clarified, the product was immersed in anhydrous ethanol for 8 h, and finally, the product was freeze-dried in a vacuum freeze-drying instrument for 48 h. Three kinds of temperature-sensitive aerogels were designated as CCNC-1NIPAM, CCNC-2NIPAM, and CCNC-3NIPAM, with a mass ratio of NIPAM to CCNC of 1:1, 1.5:1, and 2:1, respectively.

### 2.4. Characterizations

Scanning electron microscopy (SEM) images were acquired using Hitachi SU8220 (Japan) at an accelerating voltage of 10 kV.

The sample powders were analyzed on a Fourier transform infrared spectroscope (Nicolet FTIRIS10, Waltham, MA, USA), followed by mixing with KBr (1:100), and then subsequently pressed into a disk. Samples were dried overnight in a vacuum oven at 60 °C before measurements were taken.

Wide-angle X-ray diffraction (Rigaku, Tokyo, Japan) was used to evaluate the crystallinity of the samples. The measured voltage was 40 kV, and the current was 100 mA. The Segal empirical formula [Equation (1)] was used to calculate the crystallinity of cellulose (Cr. I), where *I*_002_ is the strong diffraction peak near 22.4° and *I*_am_ is the strong diffraction peak in the amorphous region (the highest diffraction intensity between *I*_002_ and *I*_110_ is used here).

(1) $$\mathrm{Cr}.\;I\;(\%)=\frac{I_{002}-I_{\mathrm{am}}}{I_{002}}\times 100\%$$

CCNCs and the CCNC-NIPAM were characterized by K-Alpha X-ray photoelectron spectroscopy (Thermo Scientific K-Alpha, Waltham, MA, USA) in the range of 0~700 eV, and the data were processed by the software provided with the device.

The compression performance of the CCNC-NIPAM was determined using a universal testing machine (AGS-X10KN, Tokyo, Japan) at a compression rate of 10 mm/min, and the rod-like sample deformation rate was set at 80% without cyclic testing.

The thermal stability and decomposition temperature of the CCNCs and CCNC-NIPAM were characterized via a thermogravimetric analysis. Then, 2~10 mg of CCNCs and CCNC-NIPAM were weighed and tested by a thermogravimetric analyzer (NETZSCH TG 209F3, Selb, Germany) under N_2_ protection. The heating rate was 10 °C/min, and the temperature range was 30~800 °C.

The size of the particle and the charge it carries on the surface of the sample in suspension (0.01 wt%) were determined at 25 °C using a nano-zetasizer device (Marvin Nano-ZS90X, Malvern, UK). The average zeta potential was recorded for 5 measurements, each consisting of 12 repeated runs.

The prepared CCNC was characterized by ADVANCE NEO 400WB NMR spectrometer (Bruker, Saarbrücken, GermanY).

### 2.5. The Yield of CCNCs

The total volume of CCNC suspension was accurately measured, and 30 mL of suspension was taken with a pipette and placed in a freeze-dried bottle that had been dried and weighed. The suspension was frozen at −40 °C and then placed in a vacuum freeze-drying instrument. After the samples were completely dried to constant weight, they were collected and weighed, and the concentration of suspension was calculated according to the weight of the CCNCs. The yield of CCNCs is calculated by Equation (2).

(2) $$\mathrm{Yield}\;(\%)=\frac{(m_{1}-m_{2})V}{30m}\times 100\%$$

where m_1_ is the total mass of the freeze-dried bottle and sample CCNCs after drying, in g; m_2_ is the mass of the pre-weighed freeze-dried bottle, in g; V is the total volume of CCNC suspension obtained, in mL; and m is the mass of bagasse cellulose treated by APS oxidation, in g.

### 2.6. The Oxidation Degree of CCNCs

In this study, conductance titration was used to determine the oxidation degree of CCNCs. Firstly, 0.2500 g of CCNC powder was added into a beaker, and 50 mL of 0.01 mol/L HCl solution was added. After mixing evenly, the CCNC suspension was titrated with 0.01 mol/L sodium hydroxide (NaOH) solution, and the volume and corresponding potential of the NaOH solution were recorded. The calculation formula for carboxyl group content in CCNCs is shown in Equation (3).

(3) $$\mathrm{DO}\;(\%)=\frac{1.62\;(V_{2}-V_{1})}{0.2500-0.36(V_{2}-V_{1})}\times 100\%$$

where V_2_ − V_1_ is the volume of NaOH solution consumed by the carboxyl group on the CCNCs, in mL.

### 2.7. The Porosity of the CCNC-NIPAM

The porosity of the CCNC-NIPAM was determined using the liquid displacement method. Ethanol is used as the replacement liquid because ethanol will not cause an expansion or contraction when permeating into the thermosensitive nanocellulose aerogel. A certain volume of freeze-dried CCNC-NIPAM was cut, its mass was accurately weighed, and the aerogel was immersed in ethanol for 24 h, then suspended for 20 s to remove excess ethanol from the surface of the aerogel and weighed. The calculation formula is as Equation (4).

(4) $$\mathrm{porosity}\;(\%)=\frac{m_{1}-m_{0}}{\rho V}\times 100\%$$

where m_1_ is the total mass of aerogel and ethanol, in g; m_0_ is the dry weight of aerogel before soaking, in g; ρ is the density of ethanol, in g/cm^3^; and V is the volume of the aerogel sample, in cm^3^.

### 2.8. Swelling and Deswelling Properties of CCNC-NIPAM

The swelling ratio (SR) of the CCNC-NIPAM was determined. A certain amount of aerogel was weighed and immersed in 30 mL of PBS solution at different temperatures (PBS, 25 °C and 39 °C, pH = 6.98) and stirred. The expanded gel was removed from the solution at regular intervals and then weighed after standing in the air for 20 s. All experiments were repeated three times. The SR is calculated by Equation (5).

(5) $$\mathrm{SR}\left(\%\right)=\frac{W_{1}-W_{0}}{W_{0}}\times 100\%$$

where W_1_ is the mass of aerogel after swelling, in g, and W_0_ is the initial mass of the aerogel, g.

CCNC-1NIPAM, CCNC-2NIPAM, and CCNC-3NIPAM, which reached equilibrium in deionized water at 25 °C, were placed in constant-temperature water at 60 °C. The gel shrunk and produced swelling changes due to heat. After removing the gel at regular intervals, the excess water was wiped off the surface of the gel with filter paper, and the gel was weighed until it was constant. The degree of detumescence is calculated by Equation (6):

(6) $$R_{W}=(m_{t}-\underset{x\to \infty }{\lim}-m_{0})/(m_{e}-m_{0})$$

(m_t_ is the gel mass measured at the time during the swelling elimination process, and m_e_ is the gel swelling equilibrium mass).

### 2.9. The Loading and Encapsulation Efficiency of CCNC-NIPAM

The spare aerogel was stirred at 500 r/min in solutions with different initial concentrations of TXM, and then the aerogel loaded with the drug was removed and lyophilized. To calculate the loading efficiency (LE) and encapsulation efficiency (EE) of the aerogel on the TXM, a series of TXM standard solutions with a concentration gradient were configured. The absorbance of the solution at 251 nm was scanned and measured using a UV–visible spectrophotometer to generate a calibration curve. The drug-loaded aerogel was ground into powder and weighed accurately. The samples were dispersed in phosphate buffers at different temperatures (PBS, 25 °C and 39 °C, pH = 6.98). After stirring at 700 r/min for 24 h, the TXM content in the supernatant was determined using the peak at 251 nm as the specific absorption peak. The LE and EE are calculated by Equations (7) and (8). All tests were repeated 3 times, and the averages were recorded.

(7) $$\mathrm{LE}\left(\%\right)=\frac{m_{0}}{m_{1}}\times 100\%$$

(8) $$\mathrm{EE}\left(\%\right)=\frac{m_{0}}{m_{2}}\times 100\%$$

where m_0_ is the TXM mass loaded on aerogel, in g; m_1_ is the aerogel mass, in g; and m_2_ is the TXM mass input in the initial drug loading solution, in g.

### 2.10. Drug Release Behavior

Drug release is a complex phenomenon that occurs through a diffusion mechanism. In this study, the CCNC-3NIPAM with the highest LE was selected and put into 200 mL of the PBS solution at 25 °C and 39 °C, respectively, for the drug release study to explore the relationship between the temperature and drug release behavior of the gel. To evenly distribute the released TXM in the PBS solution, the PBS solution was oscillated in a constant-temperature shaker at a rate of 200 r/min. Different time intervals were set, and 1.0 mL of supernatant was extracted for analysis. To keep the total solution volume constant, an equal amount of fresh solution (1.0 mL) was added. The absorbance of TXM in the solution at 251 nm was measured using a UV–visible spectrophotometer and compared with the standard curve to calculate the cumulative release of TXM. The cumulative drug release is calculated by Equation (9):

(9) $$\mathrm{Cumulative}\;\mathrm{TXM}\;\mathrm{release}\left(\%\right)=\frac{V_{e}\sum C_{i}{+V}_{0}C_{n}}{m_{\mathrm{Drug}}}\times 100\%$$

where V_e_ is the volume of solution extracted within a predetermined time interval, in mL; V_0_ is the initial volume of the solution, in mL; ∑C_i_ is the sum of drug concentrations released from sample to buffer at sampling interval 1 to n − 1, in g/mL; C_n_ is the drug concentration released at the NTH sampling interval, in g/mL; and m_Drug_ is the weight of TXM, in g.

### 2.11. The Release Kinetics of the CCNC-NIPAM

To further clarify the kinetics of CCNC-NIPAM, the xero-order model, first-order model, Higuchi model, and Korsmeyer–Peppas model Equations (10)–(13) were used to fit the drug release curve. The mechanism of TXM release from aerogel was investigated under all simulated temperature conditions.

(10) $${F=k}_{0}t$$

(11) $${F=k}_{2}t^{0.5}$$

(12) $${F=k}_{2}t^{0.5}$$

(13) $${F=k}_{3}t^{n}$$

where F is the cumulative drug release at time t and k_0_, k_1_, k_2_, and k_3_ are the release rate constants of the zero-order, first-order, Higuchi, and Korsmeyer–Peppas models, respectively. F is the drug release quantity at time t, and n is the diffusion or release index representing the drug release mechanism. The most suitable model for the experimental data is selected according to the comparative correlation coefficient.

## 3. Results

### 3.1. Characterization of CCNCs

The yield and oxidation degree (DO) of the CCNCs from the bagasse treated with the 1.00 mol/L, 1.25 mol/L, 1.50 mol/L, 1.75 mol/L, and 2.00 mol/L APS solutions for 16 h, 24 h, 32 h, 40 h, and 48 h were tested (no CCNCs were produced after 1 mol/L APS solution treatment for 16 h).

**Figure 1:**
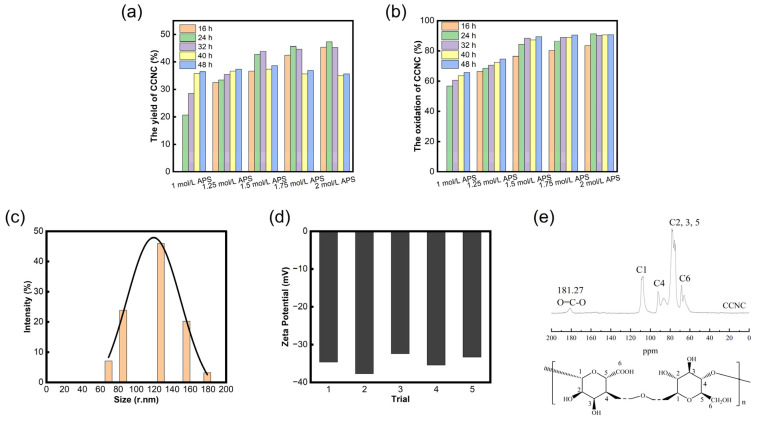
Yields of CCNCs (**a**) and oxidation degree of CCNCs (**b**) under different treatment conditions, the particle size distribution of CCNCs (**c**), and zeta potential results (**d**) of CCNCs (treated with 2 mol/L APS for 24 h; the measurement was repeated five times), and the ^13^C NMR spectra of CCNC (**e**).

Figure 1a shows that the yield of CCNCs increased and then decreased with a linear increase in APS solution concentration and oxidation time, and the highest yield was 47.32 wt%. This trend is due to the weak oxidation effect that occurs when the APS concentration is low, which is not enough to completely dissolve the amorphous region. When the concentration is too high, the oxidation degree of the bagasse is too strong, and the oxidation gradually progresses from the non-crystalline zone to the crystalline zone. In addition to oxidizing the hydroxyl group at the C6 position of the molecular chain of the bagasse, it also leads to the breakage of the reductive end of the hydrochloric glucose ring, and a side reaction of oxidation decomposition occurs [21]. Therefore, an excessive increase in the APS concentration and oxidation time will lead to a decrease in CCNC yield.

The degree of oxidation was used to evaluate the level of the oxidation reaction and the number of groups replaced on the CCNC surface. In Figure 1b, the oxidation degree (DO) of the CCNCs increases linearly with the concentration and oxidation time of APS until it becomes stable, with the highest DO reaching 90.65%. This was attributed to the ability of APS to effectively oxidize and degrade the amorphous region of the bagasse, selectively replace the primary hydroxyl group with the higher surface activity of cellulose, and introduce carboxyl functional groups. The higher the APS concentration, the longer the oxidation time and the better the oxidation effect. In addition, when the APS concentration was the same, the growth rate of the DO of the CCNCs decreased with time, which was attributed to the reduction in the number of free radicals generated by the decomposition of APS in an aqueous solution at the later stage of the reaction, leading to the slowing down of the oxidation rate and the reduction in the intensity of oxidation. The CCNCs treated with 2 mol/L APS for 24 h were selected for the subsequent characterization and preparation of aerogels.

The particle size test results of the CCNCs (treated with 2 mol/L APS for 24 h) are shown in Figure 1c. The particle size of the CCNCs provided in the software of the measuring instrument is in nanometers, mainly distributed between 60.0 and 180.0 nm, with an average particle size of 120.9 nm. The dispersion and stability of the CCNCs can be evaluated by measuring their potential in deionized water. The zeta potential results show that the average potential of the suspension of CCNCs (0.005 wt%) is −34.8 mV [Figure 1d]. At this time, there is a layer of negative charge on the surface of the CCNCs, and an electrostatic repulsion of the same charge makes the CCNC solution maintain a stable and uniform dispersion even if it is applied for a long time (three months) [22]. There are abundant active hydroxyl groups on the surface of nanocellulose without carboxylated modification, and intramolecular hydrogen bonds between groups are easy to form, leading to irregular agglomeration in deionized water. The absolute value of the negative charge on the surface of the product CCNCs increased, and its dispersion increased after the carboxylated nanomodification of the bagasse through the APS one-step oxidation method, providing the conditions for the full participation of CCNCs in the reaction. In addition, in the field of controlled release of pesticides, drug release experiments of controlled release preparations are often conducted in a phosphate buffer (PBS) solution. According to the DLVO theory [23], the stability of the solution is jointly determined by van der Waals force and electrostatic repulsion caused by double electric layers; that is, Na^+^ and K^+^ ions in the PBS solution will destroy the thickness of the double electric layer of the negatively charged solute and weaken the repulsion force between ions. Therefore, it is necessary to introduce a carboxyl group on the surface of nanocellulose to reduce the content of the active hydroxyl group.

Figure 1e shows the ^13^C NMR spectra of the CCNCs. The absorbed signals of the samples are mainly at the chemical shift range of δ = 50~120 ppm, showing a typical cellulose nuclear magnetic signal absorption peak. The absorption signals at the chemical shifts δ = 68.28, 91.85, and 107.19 ppm correspond to the crystalline regions C6, C4, and C1, while the non-crystalline regions C6 and C4 are located at δ = 65.48 and 86.76 ppm, respectively. The strong absorption peaks between δ = 70 and 81 ppm are attributed to C2, C3, and C5, which are not connected to the carbon ring by the glycoside bonds. In addition, the CCNCs showed a peak at the chemical shift δ = 181.18 ppm, corresponding to the chemical shift of the carbonyl group (C=O). The results indicated that carboxylated nanocellulose was obtained after the oxidative degradation of the CCNCs with persulfuric acid, which was mainly caused by the oxidation of the hydroxyl group on C6 by H_2_O_2_ produced by persulfuric acid decomposition in the degradation process.

### 3.2. SEM Analysis

**Figure 2:**
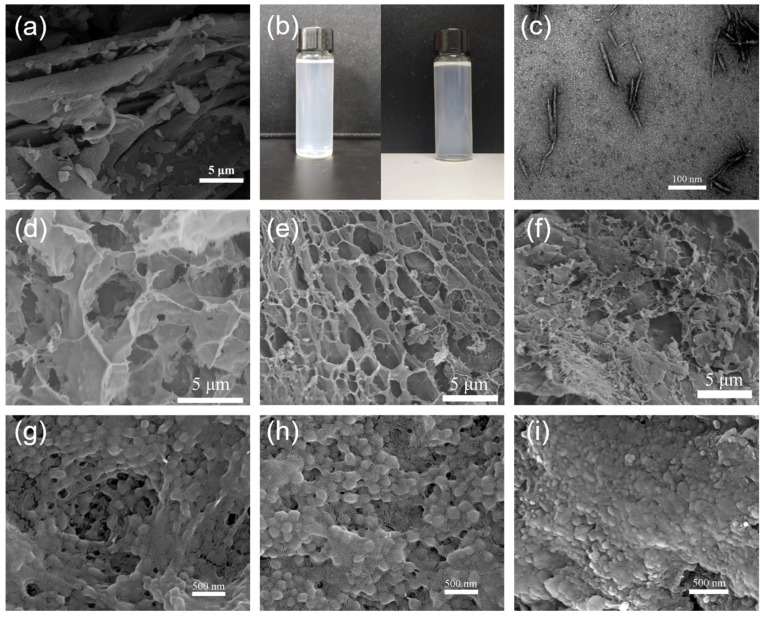
SEM images of bagasse (**a**); CCNC (**c**); CCNC-1NIPAM, CCNC-2NIPAM, and CCNC-3NIPAM prepared at 25 °C (**d**–**f**); CCNC-1NIPAM, CCNC-2NIPAM, and CCNC-3NIPAM heated at 39 °C (**g**–**i**); and a photo of CCNC suspension (**b**).

The SEM images of the bagasse Figure 2a revealed that the surfaces of the untreated bagasse had shallow surface folds and had uneven length and particle size distributions. Figure 2b shows the light blue transparent CCNC suspension after the APS treatment. The CCNC suspension was characterized by SEM, and the results are shown in Figure 2c. The CCNC suspension contains nanocrystals with uniform shape and good dispersion, suggesting that APS has a great effect on the oxidation and destruction of bagasse, and the APS one-step oxidation method successfully prepared nanocrystals with high crystallinity and low particle size.

Figure 2d–f depict the SEM images of the aerogel CCNC-1NIPAM, CCNC-2NIPAM, and CCNC-3NIPAM, in which there is no irregular network structure on the aerogel surface. With an increase in the mass ratio of NIPAM to CCNCs, the network of CCNC-NIPAMs became denser, and the average pore diameter decreased. The aerogel structure of the CCNC-1NIPAMs was loose, and the mechanical properties were poor. After freeze-drying, the CCNC-1NIPAMs could not form regular solids, because the mass fraction of the NIPAM in the CCNC-1NIPAMs was small, and the crosslinking degree of the aerogel was low. With an increase in the mass ratio of the NIPAM to the CCNCs, the network structure of the CCNC-2NIPAMs became regular, and the mechanical properties and gel stability were improved due to the increase in the number of stress points in the network. The pore size of the CCNC-3NIPAM is obviously reduced, the surface morphology is relatively compact, and the gel is more stable.

It can be concluded that the amount of NIPAM plays an important role in the structure of the aerogel, and the network structure and aperture size of the aerogel can be adjusted by changing the amount of NIPAM. The thermosensitive phase transition of CCNC-NIPAMs is caused by a change in the hydrophilic/hydrophobic balance of the cross-linked network under external conditions. Figure 2g–i show the morphologies of CCNC-1NIPAM, CCNC-2NIPAM, and CCNC-3NIPAM after heating. The hydrogen bond of the aerogel is partly destroyed with an increase in temperature, and the solvation layer of the hydrophobic part of the macromolecular chain is decomposed. At this time, the polymer changes from having a loose wire group structure to a tight colloidal structure, and the morphology changes significantly and irreversibly. In addition, the law that the network density of the series CCNC-NIPAM increases with the increasing reaction amount of NIPAM is still applicable after a temperature rise.

### 3.3. XPS Analysis

**Figure 3:**
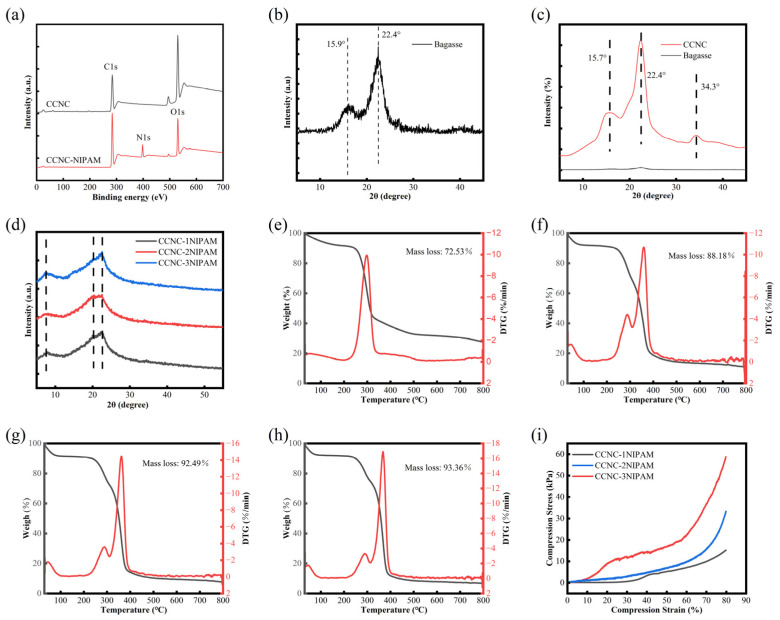
XPS spectra of CCNCs and CCNC-NIPAMs (**a**); XRD spectra of bagasse (**b**), CCNCs (**c**), CCNC-NIPAMs (**d**); TG curves of CCNCs (**e**), CCNC-1NIPAM (**f**), CCNC-2NIPAM (**g**) and CCNC-3NIPAM (**h**); stress–strain curves of CCNC-NIPAMs (**i**).

Figure 3a shows the XPS spectra of CCNCs and CCNC-NIPAMs.

XPS is an important surface analysis method that can provide element content data for a sample surface. According to the XPS spectra of the CCNCs, no signal was shown in the N1s region, which proved that there was no N element in the CCNCs. After the CCNCs were polymerized with NIPAM, the characteristic peak of nitrogen appeared at 399.1 eV in the CCNC-NIPAM spectra. The characterization results show that the N content of CCNC-NIPAM temperature-sensitive cellulose aerogels was up to 9.03% [24], and the N element existed in the form of an amino group. These highly active groups can be used to enhance the drug-loading performance of CCNC-NIPAMs and provide the necessary active sites for higher drug-loading efficiency.

### 3.4. XRD Analysis

Figure 3b–d depict the XRD patterns of the sugarcane bagasse, CCNCs and CCNC-NIPAMs. In Figure 3b, the XRD patterns of the sugarcane bagasse exhibited two strong characteristic diffraction peaks with 2θ values of near 15.9° and 21.6°, which were assigned to the (110) and (002) planes of the cellulose type I structure, respectively, and are characteristic of the cellulose type I structure. In Figure 3c, after the APS treatment, the lignin coating and hemicellulose intertwined with cellulose in the bagasse were stripped off, and a small number of new characteristic diffraction peaks appeared in the CCNCs at 34.3°, which was attributed to the exposure of the (040) surface of the cellulose type I structure [21,25]. The position of the characteristic peaks of cellulose were the same before and after the APS treatment process. However, after the APS treatment, the intensities of the characteristic diffraction peaks of the CCNCs increased by more than 1000 times compared with those before treatment, which was attributed to the disordered and loose molecular arrangement in the amorphous region of the bagasse cellulose. The APS decomposed into hydrogen peroxide and oxygen atoms under heating and acidic conditions and oxidized the reducing groups after entering the amorphous region. The partial hydrogen bond and chemical bond were destroyed, the amorphous region was dissolved, and the diffraction peak intensity increased. Before the APS modification, the crystallinity of the bagasse cellulose was low, and the spectrum burr was serious. After the modification, the spectrum of the CCNCs was smooth, which proved the increase in crystallinity. The wide peaks in the XRD results indicate that all the samples have semi-crystalline structures, and the crystallinity calculation results are provided in Table 1. The crystallinity of the bagasse was significant improved after the APS oxidation modification. The results were consistent with those of the SEM analysis Figure 2c.

Figure 3d proved that the aerogel with different NIPAM reaction quantities had three diffraction peaks at 7.5°, 20.0°, and 22.7°, and the diffraction peaks at 7.5° and 22.7° belonged to the (101) and (002) crystal planes of the cellulose, respectively. The diffraction peak at 20.0° was attributed to a large number of hydrogen bonds in the CCNC-NIPAM due to high hydrophilicity, which was the result of regular internal arrangements [26]. By comparing the XRD patterns of the CCNC-NIPAM and CCNCs, it can be found that the degree of crystallization of the CCNCs was greatly reduced. This is because, under the action of the crosslinking agent, MBA, the polymerization reaction between the CCNCs and the NIPAM monomer and the CCNCs and the NIPAM occurs to form a semi-interpenetrated aerogel network. The combination with NIPAM reduces the formation of hydrogen bonds between the CCNC molecules, destroys the compact structure of the CCNCs, and restricts the crystallization behavior of the CCNCs, resulting in a decrease in crystallinity.

### 3.5. TG Analysis

The thermal characteristics of CCNCs, CCNC-1NIPAM, CCNC-2NIPAM, and CCNC-3NIPAM were investigated and compared Figure 3e–f via TG and DTG curves. In Figure 3e, the thermal degradations of the CCNCs can be divided into three stages. The weight losses of the CCNCs at 30–100 °C were ascribed to the evaporation of adsorbed water on the sample’s surface. The CCNC decomposition process mainly occurred at 200–500 °C. The decomposition rate rapidly increased, and the highest weight loss rate reached its maximum at approximately 300 °C. With an increase in temperature (>500 °C), the CCNCs underwent the carbonization process and then decreased in decomposition rate, resulting in a total mass loss of 72.53% at the end of heating. In Figure 3f–h, The decomposition process of CCNC-1NIPAM, CCNC-2NIPAM, and CCNC-3NIPAM can be roughly divided into the following stages: The binding water inside the CCNC-NIPAM was rapidly removed at 30~200 °C, leading to weight loss. With an increase in temperature, the bulk of the CCNC-NIPAM began to decompose rapidly at 200~400 °C. The decomposition temperatures of the CCNCs were smaller than those of NIPAM, and the decomposition rate of a single CCNC reached its maximum at 300 °C [Figure 3e], which was attributed to a break in the main chain of the CCNC. When the NIPAM began to decompose, the side chain rupture of the CCNCs and the side chain decomposition of the NIPAM occurred simultaneously. Therefore, the combined decomposition of the CCNCs and NIPAM increased the maximum decomposition rate of the CCNC-NIPAM as compared to those of the CCNCs at approximately 360 °C, indicating that the NIPAM modification affected the thermal performances of the CCNCs. In the end, the CCNC-NIPAM began to carbonization and then decreased, and its decomposition rate followed a similar pattern. In addition, the TG curves [Figure 3f–h] show that the weight loss rates of the aerogels CCNC-1NIPAM, CCNC-2NIPAM, and CCNC-3NIPAM are 88.18%, 92.49%, and 93.36%, respectively, indicating that the final weight loss of the aerogel increased with an increase in the NIPAM-to-CCNC mass ratio.

### 3.6. Porosity Analysis

Table 2 lists the porosity of CCNC-1NIPAM, CCNC-2NIPAM, and CCNC-3NIPAM. The results show that with an increase in the amount of NIPAM, the porosity of the aerogel decreased from 90.29% to 85.84%. This is because the degree of cross-linking between the aerogel network and MBA increases with the amount of NIPAM reaction [27], so the density increases and the porosity decreases. This conclusion is consistent with that of the SEM characterization.

### 3.7. Compression Performance Analysis

It is highly imperative to investigate the compression properties of porous aerogel with an elastic structure, as this reflects the composition and microstructure of aerogel to a certain extent. Figure 3i shows the compressive stress–strain curves for the CCNC-NIPAMs. The compression stress of the aerogel gradually increased as the strain increased from 0 to 80%, which was attributed to the compression of the network structure of the CCNC-NIPAM from the compression strain, leading to a reduction in the elasticity and an increase in the stress of the CCNC-NIPAM. Under the same compression strain conditions, increasing the mass ratio of NIPAM to CCNC densifies the aerogel structure, resulting in an increase in the compressive stress of the aerogel. Specifically, the stress transfer caused by the increase in the number of covalent bonds between –NH_2_, –COOH, and –OH leads to an increase in the compressive stress from 15.10 kPa of CCNC-1NIPAM to 58.73 kPa of CCNC-3NIPAM.

### 3.8. FTIR Analysis

**Figure 4:**
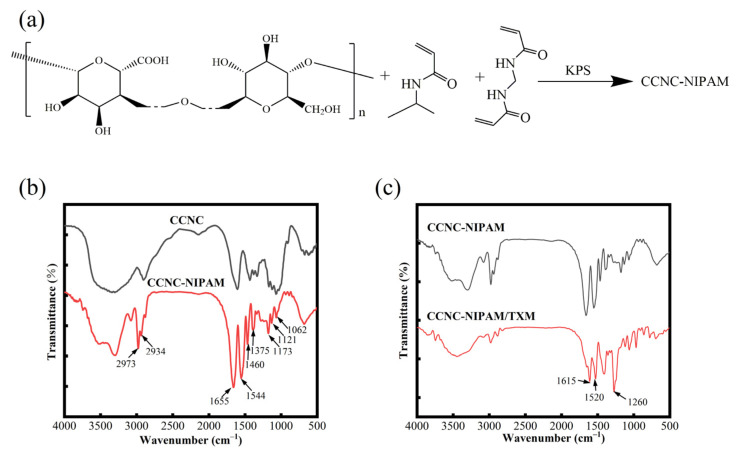
Synthesis mechanism of CCNC-NIPAM (**a**); infrared spectrum of CCNC-NIPAM and CCNC-NIPAM/TXM (**b**,**c**).

The synthesis mechanism of the CCNC-NIPAM is shown in Figure 4a, and the infrared spectra of the CCNC-NIPAM before and after drug loading (CCNC-NIPAM, CCNC-NIPAM/TXM) are shown in Figure 4b,c. In this study, three-dimensional network hydrogels were prepared through free radical polymerization using s CCNC as the substrate, NIPAM as the temperature-sensitive monomer, MBA as the crosslinking agent, and KPS as the initiator. Under the action of the acrylic group on the molecular chain of MBA, the CCNC and NIPAM have intermolecular crosslinking. In addition, the CCNC carries an active carboxyl group, which acts as a potential crosslinker and reacts with the amine group in NIPAM to form a semi-interporous network structure. 

Figure 4b shows that the characteristic peaks of the CCNC include a C–H stretching vibration peak at 2934 cm^−1^ and C–H bending and –CH_2_ bending vibration peaks at 1375 cm^−1^, 1460 cm^−1^, 1173 cm^−1^, and 1121 cm^−1^. There is a C–O–C asymmetric stretching vibration at 1062 cm^−1^, at the plane stretching peak of the C=O bond [28]. After the CCNC was cross-linked and polymerized with the NIPAM to form a semi-interpenetrating network gel, CCNC-NIPAM, the vibration peak of the methyl functional group was found at 2973 cm^−1^ [29], and the N–H bending vibration and the N–C=O stretching vibration were found at 1544 cm^−1^ and 1655 cm^−1^. The presence of these peaks indicated the successful cross-linking of the CCNC and NIPAM [30]. In Figure 4c, the C=N tensile vibration peak of CCNC-NIPAM/TXM at 1615 cm^−1^, the absorption characteristic peak of –NO_2_ at 1520 cm^−1^, and the unsaturated sulfide bond peak at 1260 cm^−1^ appeared. These characteristic peaks were attributed to TXM, indicating that TXM was successfully loaded on the aerogel.

### 3.9. Swelling and Deswelling Property of CCNC-NIPAM

**Figure 5:**
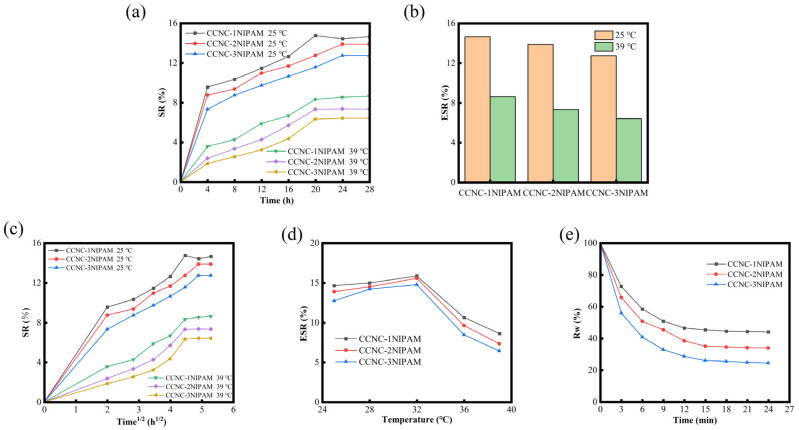
The swelling rate (**a**,**c**) and equilibrium swelling ratio of aerogel under different conditions (**b**,**d**); the deswelling kinetics curves of CCNC-NIPAM at 55 °C (**e**).

Generally, the LCST of NIPAM is 32 °C. On this basis, the swelling degree (a), swelling rate (c), and equilibrium swelling degree (b) of the CCNC-NIPAM in a temperature range of 25–39 °C under different conditions were studied, as well as the changes in the equilibrium swelling rate of the aerogel in a PBS solution with increasing temperature (d), as shown in Figure 5.

The swelling ratio (SR) of the CCNC-NIPAM in the solutions at different temperatures first increased and then gradually stabilized over time Figure 5a. Many researchers have proposed that the swelling mechanism of a polymer network is not only the simple diffusion of water molecules but also three continuous processes: water molecule diffusion into the gel network, polymer chain relaxation due to hydration, and gel network expansion in the solution. According to the relationship between the square root of the quantitative meter SR and the swelling time (t^1/2^), if the swelling process of the material takes the diffusion of water molecules as the leading step, the SR is proportional to t^1/2^; otherwise, the relationship curve between SR and t^1/2^ is S-shaped. According to the calculated results [Figure 5c], the swelling process of each CCNC-NIPAM takes the second and third stages as the control steps. Figure 5b shows the equilibrium swelling rate (ESR) of each aerogel at different temperatures. The results show that the ESR of the CCNC-NIPAM at 25 °C is higher than that at 39 °C, which reflects the temperature-sensitive property of the aerogel [31]. The phase transition temperature of the gel can be obtained from the tangential curve of the equilibrium swelling curve of the gel. At this temperature, the slope change of the swelling curve is most obvious; that is, the volume change rate is the largest. To further discuss the thermosensitive characteristics of aerogel, the ESR test of aerogel with temperature as the variable (25~39 °C) was designed [Figure 5d]. Among them, CCNC-1NIPAM, CCNC-2NIPAM, and CCNC-3NIPAM all showed the largest slope change at 32 °C, so 32 °C was taken as the LCST of the CCNC-NIPAM. In the whole temperature range, when the temperature is lower than 32 °C, the ESR of aerogel gradually increases with increasing temperature. When the temperature is higher than the LCST, the ESR decreases rapidly with increasing temperature. In addition, according to the swelling performance of each aerogel, a rule can be summarized. The higher the NIPAM reaction amount in the raw material, the lower the SR and final ESR in the process. This conclusion can be attributed to the increase in the NIPAM reaction amount, the increase in the aerogel crosslinking degree, the decrease in porosity, and the denser structure. The swelling behavior of the CCNC-NIPAM was hindered [27].

Figure 5e shows the deswelling kinetics curves of the CCNC-NIPAM at 55 °C after reaching the swelling equilibrium at 25 °C. The three gels shrink sharply within the first 5 min, and then the contraction rate slows down and reaches the contraction equilibrium after around 15 min. The contraction range increases with an increase in the NIPAM fraction. When the temperature is 55 °C, it is already higher than the volume phase transition temperature of the CCNC-NIPAM. The gel in swelling equilibrium immediately shrinks rapidly, and a shrinkage layer of the polymer chain is formed on the surface of the gel, which inhibits the penetration of solvent from the inside of the gel, and the gel shrinkage becomes slow. When the pressure gradually accumulates, the solvent inside the gel flows out, and the gel continues to shrink.

### 3.10. Load Efficiency (LE) and Encapsulation Efficiency (EE)

The initial concentration of the drug-loaded solution was set as 30 wt%, 50 wt%, and 70 wt% (compared with the mass of the CCNC-NIPAM). The test results of the drug-loaded solution are shown in Table 3. There were various non-covalent bonds between TXM and the CCNC-NIPAM, such as electrostatic attraction, hydrogen bonds, and a van der Waals force. It could be seen that with an increase in the NIPAM reaction amount, the drug loading of the aerogel also increased, up to 23.00 wt%, which was attributed to the increase in amino active sites introduced by the NIPAM and the enhanced binding ability of the drug. In addition, the drug loading and encapsulation efficiency of the aerogel are also related to the initial concentration of the drug. In general, they increase with an increase in the initial concentration and become stable until it approaches the maximum encapsulation rate. 

### 3.11. Drug Release Behavior Analysis

**Figure 6:**
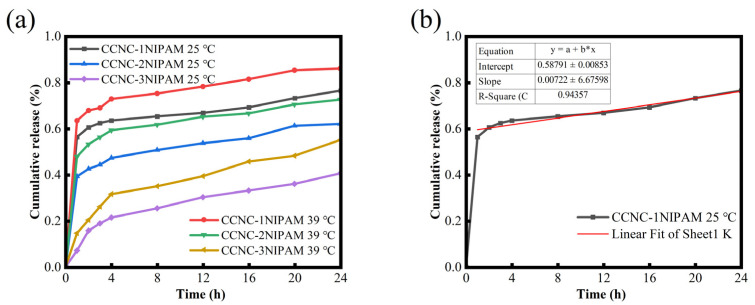
Drug release curve of aerogel within 24 h (**a**) and model fitting example diagram (**b**).

Figure 6a shows the drug release behavior of the CCNC-NIPAM at 25 °C and 39 °C.

At different temperatures, the cumulative release rate of the TXM-loaded aerogel in the PBS solution gradually increased over time. The initial burst effect of the CCNC-NIPAM in Figure 6a was attributed to the fact that part of the drug was absorbed in the pores on the surface of the CCNC-NIPAM during the drug-loading process, where the drug quickly entered the PBS solution through simple diffusion. Moreover, 120 min after the explosive release, the drug release rate of the CCNC-NIPAM decreased, and the TXM dissolved from the carrier substrate and diffused through the three-dimensional network structure in this stage to achieve drug control and continuous release [32]. At 25 °C, the cumulative release rate of CCNC-1NIPAM within 24 h reached 76.46 wt%, while that of CCNC-3NIPAM was only 40.55 wt%. Also, at 39 °C, the cumulative release rate of CCNC-1NIPAM within 24 h reached 86.10 wt%, while that of CCNC-3NIPAM was only 55.06 wt%. This is because with an increase in the NIPAM reaction amount, the increase in the number of amino active sites leads to an enhancement of the ability of aerogel to bind to drug molecules, a decrease in the drug molecule release rate, and an extension in the drug molecule release time. In addition, as the amount of NIPAM increased, the network structure of the CCNC-NIPAM densified, and the network structure hindered drug release to a certain extent.

In Figure 6a, the drug release behavior of the CCNC-NIPAM temperature-sensitive nanocellulose-based aerogel was also summarized as a function of temperature. The results showed that under the same reaction amount of NIPAM, the drug release rate of the aerogel at 39 °C was significantly higher than that at 25 °C, which was attributed to the LCST of the NIPAM being 32 °C. At 25 °C (lower than the LCST), the polymer chain of the NIPAM unfolded, but the inherent aerogel structure did not allow the polymer chain to be completely disintegrated, so a hydrophobic environment was formed in the gel, resulting in TXM aggregation, which was not easy to dissolve and diffuse through PBS solution, and the detected drug release rate decreased. When the temperature rose to 39 °C, the polymer chain of the NIPAM tended to be coiled, and the 3D network contraction of the gel squeezed the internal liquid, and the release rate of TXM increased under the combined action of pressure and concentration difference [29]. The above results proved that the CCNC-NIPAM had good temperature responsiveness, and the controlled release performance of the CCNC-NIPAM could be changed by adjusting its network structure.

### 3.12. Analysis of Fitting Results of Release Kinetics

Figure 6b shows the fitting diagram of the drug release curves of the CCNC-NIPAM using the zero-order model. The zero-order, first-order kinetics, Higuchi, and Korsmeyer–Peppas models were used to fit the drug release curves of the CCNC-NIPAM under different conditions. The fitting results (Table 4) show that the slow-release kinetic fitting curves are more consistent with the first-order kinetic model and the Korsmeyer–Peppas model, with an R^2^ greater than 0.9444. In the Korsmeyer–Peppas model, the parameter “*n*” is an important parameter for the release mechanism. When n is less than 0.45, the release mechanism of the CCNC-NIPAM is Fick diffusion. When 0.45 < *n* < 0.89, the release mechanism of the CCNC-NIPAM is non-Fick diffusion. When *n* > 0.89, drug release is mainly due to the erosion of the carrier skeleton. In this study, the fitting parameter, “*n*”, of aerogel drug release was always less than 0.45, indicating that the drug release in the CCNC-NIPAM was mainly based on the diffusion mechanism.

## 4. Conclusions

In this study, CCNCs were successfully prepared from bagasse, and the temperature-sensitive monomer NIPAM was introduced to prepare a CCNC-NIPAM, which was applied in the field of pesticide controlled release. The ammonium persulfate one-step oxidation method has the characteristics of using low-purity materials, having a simple operation, and using high-crystallinity products. The average particle size of the prepared CCNCs is 120.9 nm, and the average surface potential of the CCNCs is −34.8 mV. The porosity of the CCNC-NIPAM exceeded 85%, with a highly porous structure and rich functional groups. After loading the model drug, TXM, the CCNC-NIPAM became an ideal drug release carrier and showed obvious temperature responsiveness. With the increasing mass ratio of NIPAM to CCNCs, the degree of cross-linking of CCNC-NIPAM increased, which changed the mechanical properties and porosity and also had a certain effect on the swelling behavior, and the cumulative release rate of TXM decreased during the same time. Specifically, at 25 °C, the cumulative release rate of CCNC-1NIPAM reached 76.46 wt% within 24 h, while that of CCNC-3NIPAM was only 40.55 wt%. At 39 °C, the cumulative release rate of CCNC-1NIPAM reached 86.10 wt% within 24 h, while that of CCNC-3NIPAM was only 55.06 wt%. The results show that the release kinetic fitting curves are more consistent with the first-order kinetic model and the Korsmeyer–Peppas model. The purpose of this study was to explore a way to make full use of solid waste bagasse and to provide a reference for research on TXM delivery formulas.

## Figures and Tables

**Table 1:** Crystallinity of each sample in the XRD pattern.

Sample	I_002_	I_am_	Cr. I (%)
bagasse	60	20	50.0
CCNC	5591	2080	62.8

**Table 2:** Aerogel porosity analysis.

Sample	Porosity (%)
CCNC-1NIPAM	90.29
CCNC-2NIPAM	87.23
CCNC-3NIPAM	85.84

**Table 3:** The drug loading and encapsulation efficiency of aerogel under different NIPAM dosages.

Sample		30 wt%	50 wt%	70 wt%
CCNC-1NIPAM	LE (%)	14.57	20.23	20.56
	EE (%)	48.57	40.46	29.37
CCNC-2NIPAM	LE (%)	15.93	21.48	21.99
	EE (%)	53.10	42.96	31.41
CCNC-3NIPAM	LE (%)	16.32	22.57	23.00
	EE (%)	54.40	45.14	32.86

**Table 4:** Release kinetics equations and coefficient correlation values based on the release profile of TXM encapsulated in hydrogels.

Kinetic Models	Coefficient Correlation	CCNC-1NIPAM		CCNC-2NIPAM		CCNC-3NIPAM	
		25 °C	39 °C	25 °C	39 °C	25 °C	39 °C
Zero-order	k_0_	0.0072	0.0091	0.0094	0.0092	0.0116	0.0153
	R^2^	0.9436	0.9368	0.9543	0.8989	0.9359	0.9266
First-order	k_1_	0.0225	0.0405	0.0197	0.0245	0.0160	0.0245
	R^2^	0.9550	0.9762	0.9715	0.9444	0.9596	0.9590
Korsmeyer -Peppas	k_2_ (*n*)	0.0936	0.0863	0.1123	0.1496	0.4051	0.3829
	R^2^	0.9955	0.9840	0.9993	0.9993	0.9755	0.9729
Higuchi	k_3_	0.0433	0.0557	0.0596	0.0566	0.0712	0.0933
	R^2^	0.9514	0.9810	0.9868	0.9609	0.9827	0.9720

## Data Availability

The data presented in this study are available on request from the corresponding author.
